# Dual-Silane Premodified Silica Nanoparticles—Synthesis and Interplay between Chemical, Mechanical, and Curing Properties of Silica–Rubber Nanocomposites: Application to Tire Tread Compounds

**DOI:** 10.1021/acsomega.2c00665

**Published:** 2022-05-18

**Authors:** Enzo Moretto, João P. C. Fernandes, Mariapaola Staropoli, Vincent Rogé, Pascal Steiner, Benoît Duez, Damien Lenoble, Jean-Sébastien Thomann

**Affiliations:** † MRT Department, Luxembourg Institute of Science and Technology, 41 rue du Brill, L-4422, Belvaux, Luxembourg; ‡ Goodyear S.A, Avenue Gordon Smith, L-7750, Colmar-Berg, Luxembourg

## Abstract

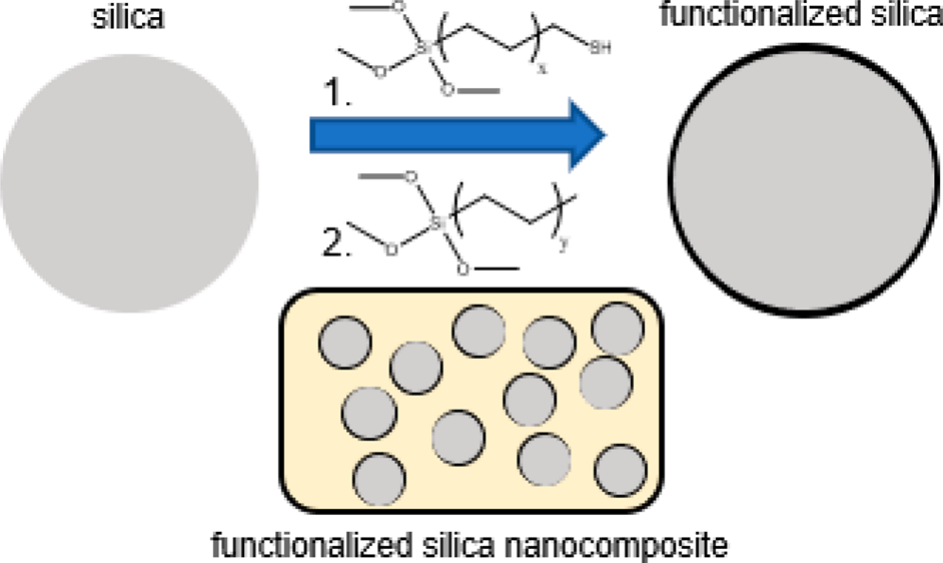

In silica–rubber based nanocomposites, a single organo-silicon is often used to compatibilize and covalently link silica to rubber. In this work, we have investigated the impact, at micro- and macroscales, of the decoupling of the hydrophobization and the coupling activity of silane by pretreating silica with two different silane chemistries. The first one, a mercaptosilane, is the coupling agent that promotes a covalent link between silica and rubber during the sulfur-mediated vulcanization reaction. The second one, an alkylsilane, aims to improve the silica dispersion. For both kind of silanes, we have varied the chain length and studied at macroscale the dynamic mechanical properties through the key indicators that are *E*′′ as loss modulus, *E*′ as storage modulus, and their respective ratio tan δ. The shorter silanes combination yielded an improvement in terms of wet grip indicators with tan δ at 0 °C increasing from 0.205 to 0.237 while maintaining rolling resistance indicators at the same level. We have evaluated the impact of the silane chemistry onto the cross-linking reactivity within the fabricated rubber-based nanocomposites by using moving-dye rheometer measurements (MDR). By purposely using atomic force microscopy (AFM), we have studied the silica dispersion in the matrix and the rubber/silica interface and provided the rationale explanation of the mechanical properties observed at the macroscale. AFM observation pointed out the existence of a soft interface around silica fillers when long alkylsilanes were used. We infer that this interface impacts the polymer–filler dynamic and subsequently affects the mechanical properties of the composite material.

## Introduction

Historically, tire rubber compounds were reinforced using carbon black. The good interaction between this filler and the usual polymers matrices implies very good performances as well as an easy mixing of the filler with the polymer blend. Later, silica was introduced as a potent filler, but due to its hydrophilic nature, new strategies had to be found to compatibilize the particles with the matrix to reach desirable performances. Organosilanes have been the first choice as they allow covalent bonding of silica with the polymer and hydrophobize the surface of the particles, inducing a much better dispersion and reinforcement of structure. Since then, researchers have been working on these two filler properties and investigating various strategies to improve performances, using new materials and processes. The *in situ* growth of silica particles has proven to be an efficient way to achieve a very high dispersion of such particles into the matrix.^1,2^ Other works investigated the use of nonsilane coupling agents.^3,4^ Beyond silica and carbon black, innovative alternative fillers such as silicon particle and graphene oxide are developed.^5^ With the growing need to find sustainable filler materials, research in the fillers domain becomes driven by the challenging task to make new fillers cope with previous technology performance levels.^6^ The filler morphology is another important research topic, and extensive work is being conducted on the use of anisotropic fillers^7−10^ based on silica, clay, or cellulose nanocrystals. Other strategies, not focusing on the filler itself, work around the opportunity to valorize waste as a usable resource for the tire industry.^11^ Tire-tread compounds face a challenge in which wet-grip performances need to be sustained, while improving rolling resistance in order to comply with new environmental regulations and original equipment manufacturers requirements.^12^ The introduction of silica as a filler opened the door of progress for wet traction while decreasing significantly rolling resistance, even it was mainly the use of silanes that really enabled it.^13^ By creating an interface between the silica particles and the polymer matrix, silanes modify interactions and forces transmission during the mechanical solicitation of the material. Usually, silanes are added into the rubber blend during the mixing of the polymer, silica, and other ingredients. It allows for a simple and effective way to incorporate them. Another strategy is the pretreatment of the silica particles before their addition. This method enables a better control of the silanization and finer tuning of the final material properties, as well as helping to avoid alcohol production in the blend when silanes react. This method of processing rubber is safer and simplified. The idea of multiple silanes on silica to bring out new properties is not recent,^14^ and has been already been proven to be an efficient way to solve the aggregation issue in bitumen filled silica.^15^ But according the best of our knowledge, dual-silane silica has not been applied to tire tread rubber. Our work on presilanized silica stands within this context. Silanes are used with silica not only as binding agent with the rubber matrix, but also as antiflocculent ingredients which increase the silica dispersibility in the polymer.^16^ Indeed, silica is a mineral hydrophilic filler and tends to aggregate through hydrogen bonding when mixed with polymers. Silane molecules, when reacting with hydroxyl groups on the silica surface, turn the silica hydrophilic surface into a hydrophobic one, hence increasing compatibility with the rubber matrix.^17,18^ Bis-triethoxysilylpropyltetrasulfide (TESPT) for example is a widely used silane, as it plays the role of coupling agent between silica and rubber through its tetra sulfide bond and participates in the hydrophobization of the filler.^19,20^ Another aspect of the silanization of silica particles is that it affects the vulcanization behavior of a rubber compound and more specifically the cross-link density and the scorch time.^18,21−23^ The concept of scorch time is essential when it comes to rubber compounding as it describes the time window during which the green compound is still shapeable before becoming irreversibly rubbery. The introduction of silanes in the system changes the cure dynamic by affecting the molecular mobility within the matrix. It also affects the cross-link density, as mercaptosilanes can react with the polymer chains and bridge them with silica particles or other polymer chains. Many studies worked out the impact of silanes on rubber compounds, especially the effect of silanes on rubber cross-linking and silica dispersion.^24,25^ Usually, only one silane is used in the compound, or different silanes are compared one-to-one in order to understand the impact of their structures on the system.^18,26,27^ In this work, we investigate the idea of a dual-silane presilanized silica, where two different silanes are grafted onto silica particles. For this matter, we used a method that we call “base catalyst pre-loading”, to achieve high silica coverage of silane without creating multiple layers on particles. As silanes promote the silica binding to the polymeric matrix as well as the dispersion of particles into the polymer,^24,25^ we want to tailor silica surfaces with two different silanes populations. A first silane plays the role of coupling agent by chemically bridging silica and rubber. The second acts as a hydrophobicity enhancer for the silica particles and mitigates the coupling reaction of silica with rubber. Indeed, severe silica aggregation can occur with silanes bridging themselves when polymerizing together due to their chemical structure and reactivity. For this matter, we study here how the respective molecular size of the two silanes affects each other. By combining alkylsilanes with mercaptosilanes, we investigate the possibility of mitigating the coupling silane reactivity by means of steric hindrance to open new and original chemical routes aiming at improving the cure behavior and the mechanical properties of the rubber-based nanocomposite.

## Experimental Section

**Figure 1:**
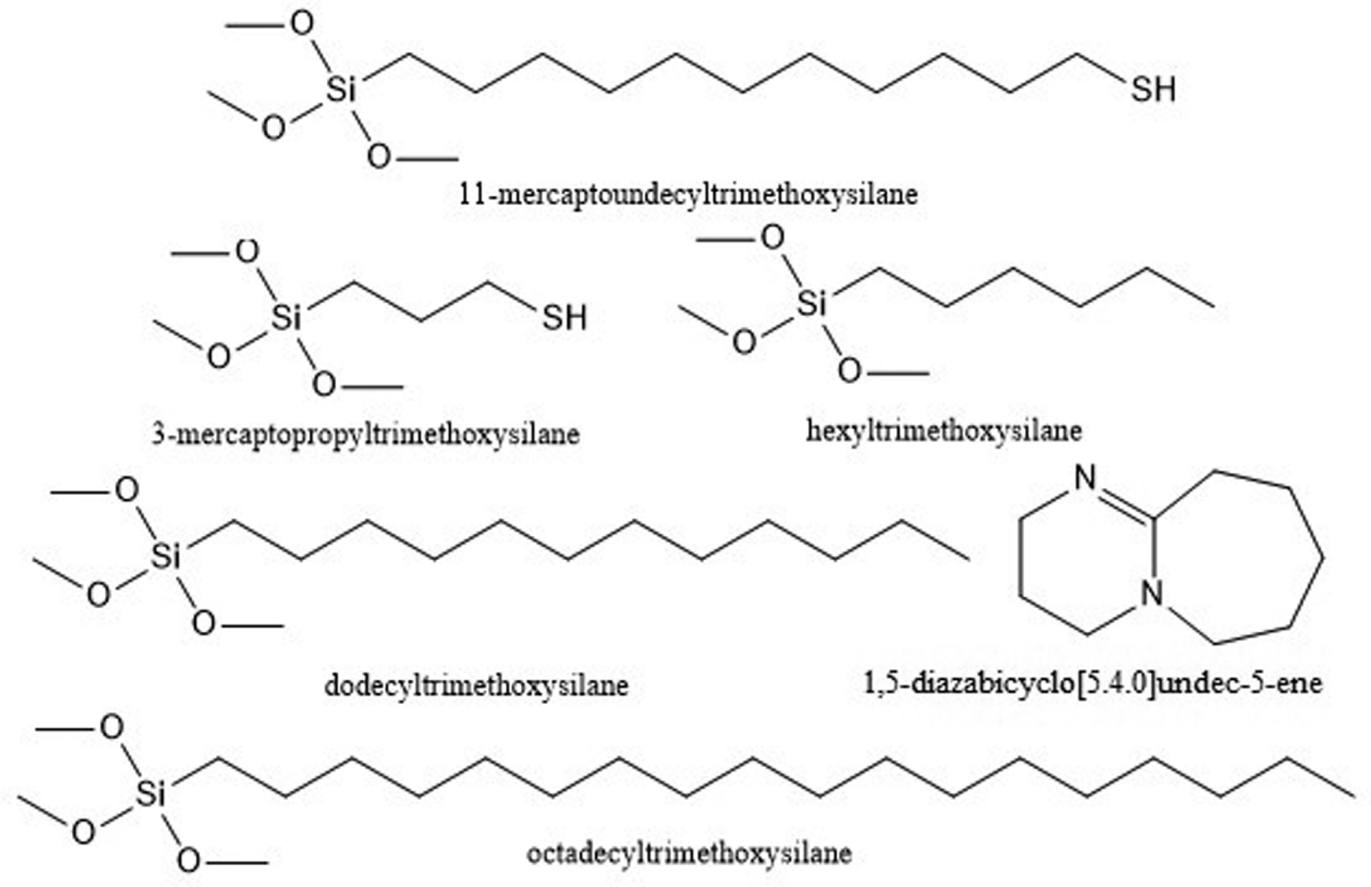
Chemical structures of silanes and base catalyst.

For this study, high dispersibility silica (HDS) was used. This silica is obtained industrially by a wet precipitation process which results in 10 nm primary particles size with a specific surface area of 200 m^2^/g (see Figures S1 and S2 in Supporting Information). An infrared spectrum of this silica is provided in Figure S3. 3-Mercaptopropyltrimethoxysilane (MPTS), 11-mercaptoundecyltrimethoxysilane (MUTS), hexyltrimethoxysilane (C6), dodecyltrimethoxysilane (C12), and octadecyltrimethoxysilane (C18) were supplied by Gelest. Synthesis grade toluene, THF, and 1,5-diazabicyclo [5.4.0] undec-5-ene (DBU) were supplied by Sigma-Aldrich. The chemical structures of silanes and DBU are shown in Figure 1.

### Dual-Silane Premodified Silica Synthesis

A typical presilanization is performed in three steps. First the base catalyst is preloaded as follows: 30 g of HDS silica are suspended in 500 mL of a solution of 0.34 g/L of DBU in THF under stirring at room temperature for 10 min. The silica is then separated by centrifugation and dried under reduced pressure at room temperature. In the second step, 30 g of DBU-treated silica are suspended in 167 mL of toluene. The suspension is heated up to 110 °C and 0.023 mol of MPTS or MUTS are added. The reaction is carried at 110 °C under stirring for 24 h. Then silica is separated and rinsed with fresh toluene and centrifugation. The silica washing operation is repeated three times to ensure that no unreacted silane is left on the silica. In the third step, 30 g of the previously modified silica are suspended in 167 mL of toluene. The suspension is heated up to 110 °C and 0.023 mol of the desired alkylsilane are added. The reaction is carried at 110 °C under stirring for 24 h. Then silica is separated by centrifugation and cleaned with fresh toluene. This operation is also repeated three times to ensure that no unreacted silane is left on the silica. Finally, the dual-silane premodified silica particles are dried under vacuum at room temperature and stored in a glass container for later use.

### Silica Mixing in Rubber Matrix

Polystyrene-butadiene (SBR, solution SBR, 21% styrene, 50% vinyl) polybutadiene rubber (BR, neodymium catalyzed polybutadiene), treated distillate aromatic extracted (TDAE) lubricating oil, zinc oxide, stearic acid, *N*-(1,3-dimethylbutyl)-*N*′-phenyl-*p*-phenylenediamine (6-PPD), sulfur, 2-mercaptobenzothaizole (MBT), diphenylguanidine (DPG), and *N*-cyclohexyl-2-benzothiazolesulfenamide (CBS) have been used. At each mixing step ingredients have been mixed using a HAAKE PolyLab QC ThermoScientific internal mixer, and then the compound was further mixed in a roll-mill. Component quantities and mixing steps have been summed up in the Table 2. All green composite materials have been cured in a hydraulic press at 170 °C for 10 min under a pressure of 150 kPa or in a moving-dye rheometer (MDR) measuring device for the curing behavior measurements. Table 1 below shows the correspondence between compounds names and silanes grafted onto silica.

**Table 1:** - Silanes/Compounds Correspondence Table

compounds names	silane 1	silane 2
reference HDS silica	N/A	N/A
C3SH+C6	3-mercaptopropyltrimethoxysilane	hexyltrimethoxysilane
C3SH+C12	3-mercaptopropyltrimethoxysilane	dodecyltrimethoxysilane
C3SH+C18	3-mercaptopropyltrimethoxysilane	octadecyltrimethoxysilane
C11SH+C6	11-mercaptoundecyltrimethoxysilane	hexyltrimethoxysilane

**Table 2:** Composition and Mixing Steps of Compounds

components	phr	mixing conditions
Step 1		
polystyrene-butadiene	80	80 °C for 10 min
polybutadiene	20	
TDAE oil	25	
zinc oxide	0.5	
stearic acid	3	
dual-silane silica	65	
Step 2		
Step 1 compound		80 °C for 7 min
dual-silane silica	15	
6PPD	2.5	
Step 3		
Step 2 compound		60 °C for 1 min 45 sec
zinc oxide	2	
sulfur	1.1	
MBT	0.3	
DPG	3.2	
CBS	2.3	

### Characterization

Dual-silane premodified silica fillers have been characterized by thermogravimetric analysis (TGA) and solid state nuclear magnetic resonance of silicon 29 (^29^Si ssNMR). Silane loading was quantified by TGA as followed: 10 to 20 mg of silica powder was placed in an alumina crucible and heated from 25 to 1000 °C at 10 °C/min. An empty alumina crucible is also placed in the furnace for reference. Solid-state ^Si^ MAS (Magic Angle Spinning) NMR spectra were acquired on a Bruker Avance 400 MHz spectrometer (9.4 T wide bore magnet) equipped with a 4 mm BL4 X/Y/H probe. Magic angle spinning was performed at 6.5 kHz using ceramic zirconia rotors of 4 mm in diameter. The signal of talc was used to calibrate the silicon chemical shift scale (−98 ppm). Acquisition parameters used were the following: a spectral width of 300 ppm, a 90° pulse length of 4.5 μs, an acquisition time of 15 ms, a recycle delay time of 60 s, and about 3000 accumulations (48 h). High power proton dipolar decoupling during the acquisition time was set to 70 kHz. Solid-state ^29^Si MAS (magic angle spinning) NMR spectra were also acquired on an Agilent VNMRS DirectDrive 400 MHz spectrometer (9.4 T wide bore magnet) equipped with a T3HX 3.2 mm probe. Magic angle spinning was performed at 6.5 kHz using ceramic zirconia rotors of 3.2 mm in diameter, the signal of talc was used to calibrate the silicon chemical shift scale (−98 ppm). Acquisition parameters used were the following: a spectral width of 300 ppm, a 90° pulse length of 5 μs, an acquisition time of 15 ms, a recycle delay time of 60 s, and about 4400 accumulations (72 h). High power proton dipolar decoupling during the acquisition time was set to 70 kHz. Mechanical properties of the resulting composite materials have been tested by dynamic mechanical analysis (DMA) at 1 and 10 Hz, a free length of 5 mm and a temperature sweep from −80 °C up to 100 °C. The curing behavior of the green compounds was evaluated by MDR with a MDR 2000 rheometer from Alpha Technologies, at a frequency of 1.667 Hz, a strain of 0.5 degree, at a temperature of 160 °C during 60 min. Sample dimensions are 43 mm in diameter and 2 mm thickness. Atomic force microscopy images of cryo-ultramicrotomed surfaces of the samples were acquired using the AM-FM mode of the MFP-3D Infinity AFM instrument (Asylum Research). All measurements were made under ambient conditions, and a standard cantilever holder for operation in air was used. Images of 10 × 10 μm^2^, 5 × 5 μm^2^, and 2 × 2 μm^2^ areas were taken with a resolution of 256 × 256 pixels at a scan rate of 1 Hz. Cantilevers’ spring constants used in this study were about 30 N/m (AC160TS-R3 model from Olympus). The first and second resonant frequencies for AC160TS-R3 cantilevers were about 300 kHz and 1.6 MHz, respectively. To ensure repulsive intermittent contact mode, the amplitude set point was adjusted so that the phase is well fixed below 90°. This allows for the acquisition of complementary stiffness contrast images simultaneously with topography.

## Results and Discussion

### Synthesis of the Dual-Silane Silica Particles and Their Characterization

The main objective of the synthesis method is to increase the yield of silane grafting on silica particles without falling into a multilayer regime. Consequently, experimental conditions focused on promoting a silica–silane surface reaction and preventing the oligomerization of silane in the solvent. First, silica particles are loaded with the base catalyst DBU to increase reactivity on the surface. DBU has a strong p*K*_a_ (∼13.5 in water^28,29^). Thus, DBU may lead to substantially deprotonate hydroxyl groups of the silica surface due to the p*K*_a_ difference. If so, the silica surface should be more prone to react with silanes. The presence of an amine catalyst also improves the grafting yield and the stability of the silane–silica bond.^30^ Second, the reaction is carried in toluene, as it solubilizes silanes and has relatively a high boiling point compared to another hydrocarbon solvent. Also, toluene is a good middle ground when it comes to the formation of hydrophobic monolayer on silica, as it naturally solubilizes enough water in order to induce silanol formation from alkoxysilane, without promoting polymerization of the alkoxysilane by solubilizing too much water.^31^

**Figure 2:**
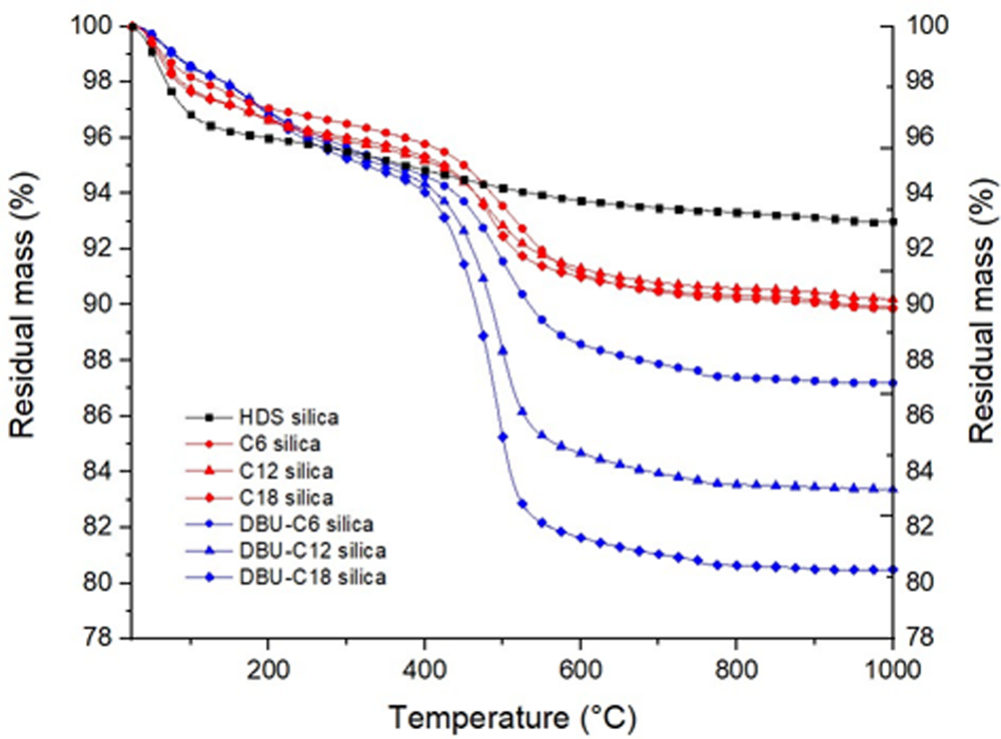
TGA curves of silanized silica (C6, C12, and C18), with and without DBU preloading.

**Figure 3:**
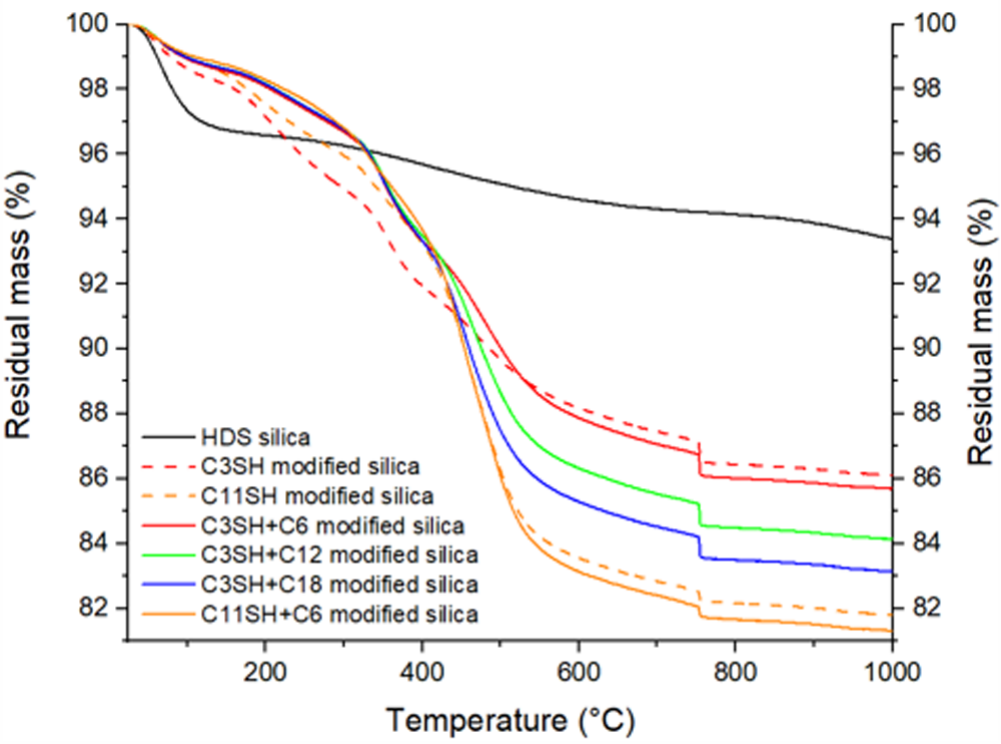
TGA curves for dual-silane premodified silica.

We can observe a significant difference of the residual mass below 130 °C when considering the thermograms of Figure 3. In the case of the HDS silica, the mass loss is attributed to the desorption of water from the silica surface. In the case of modified silica, we attribute the mass loss to the loss of the remaining alkoxy groups originating from the grafted silanes. This difference originates from the fact that HDS silica is a precipitated silica, hence featured by the absence of alkoxy groups, contrary to our modified silica for which we used methoxysilanes. Also, the initially adsorbed water on the unmodified silica is lost through the silanization and drying steps being performed to obtain dual silane modified silica. Surface hydroxyls groups Si–OH have a key role, and it has been documented^32^ that silica reactivity toward alkoxysilanes is enabled by the presence of silanols at the surface. Silica’s silanols undergo a condensation reaction with silane’s silanols, form a Si–O–Si bond, and covalently bond the silane to the silica. Prior to the condensation step, silanes must be hydrolyzed from the alkoxy form to silanols Si–OH. Since the HDS silica is produced via a wet precipitation process, silanols groups Si–OH feature the surface of such particles. An infrared spectrum of the HDS silica used in this study is shown in Figure S3 of the Supporting Information and further highlights the presence of Si–OH. The physical interaction between silanes and silica is also an important parameter, especially through hydrogen bonding of silanes onto silica. The capability of thiols to form hydrogen bonds has been questioned for a long time with few studies to shed light on this phenomenon. This is of major consideration especially in biology as sulfur and thiols are important constituents of many molecules. Some recent experimental and computational works have brought more understanding on the topic.^33,34^ According to the best of our knowledge, the interaction of a thiol–silane with silica through hydrogen bonding has not been reported and studied, unlike the very well-known interaction of amine–silane with silica.^35^ Due to the lower electronegativity difference of the sulfur–hydrogen bond (Δ ∼ 0.38) compared to the nitrogen–hydrogen bond (Δ ∼ 0.84) or oxygen–hydrogen bond (Δ ∼ 1.24), the strength of the hydrogen bond involving thiol is expected to be weak. In the case of back-bonding of the silane to the silica via hydrogen bonding, we could expect to have residual silane weakly bonded to the silica remaining after the reaction. Another critical aspect is the potential interaction between DBU and the mercaptosilane during silanization, and the possibility to deprotonate the terminal thiol of this silane. It has been reported that the p*K*_a_ of DBU is about 13.5,^28,29^ and the p*K*_a_ of an aliphatic thiol is around 11.^36^ For the p*K*_a_ of silanols, various values have been reported ranging from 5 to 9.5.^37^ The difficulty of determining the p*K*_a_ of silanols comes from the variety of spatial conformation of silanols, such as geminal, vicinal, and isolated or H-bonded silanols. All these parameters impact the acidity of the silanols. We can affirm that DBU will first deprotonate the most acidic silanols, then the less acidic ones, and finally eventually the thiol group of the silane. This is the reason why we did not mix as such DBU and the silane in the silanization media. We preferred an approach in which we deprotonated the silica’s silanols by premixing DBU and silica in a first step, then silanization is carried out as described in the Experimental Section. To assess the amount of silane grafted onto silica, the mass loss associated with the silane pyrolysis is recorded via TGA. The mass loss is calculated by subtracting the residual mass loss value at 600 °C from the value at 125 °C to isolate the silane contribution to the total mass loss of the sample. This method of calculation is inspired from the work of Kunc et al.^38^ TGA thermograms in Figure 2 and values in Table 3 show the difference between no catalysis and DBU preloading on silica. For the system without any base catalyst preloading, all silanes yield about 3.5 to 4% of mass-loss, whereas DBU preloaded silica displays a much higher grafting yield of respectively 6.95%, 10.85%, and 13.89% for C6, C12, and C18. The use of DBU clearly yields a higher silane loading on silica. The silane loading for dual-silane pretreated silica has also been characterized by thermogravimetric analysis (Figure 3) and 29Si ssNMR (Figure 5).

**Table 3:** TGA Values for Modified Silica with and without DBU Preloading

sample name	residual mass at 125 °C (%)	residual mass at 600 °C (%)	mass loss (%)
HDS silica	96.44	93.74	2.7
C6 silica	97.88	91.13	4.05
C12 silica	97.43	91.31	3.42
C18 silica	97.37	91	3.67
DBU-C6 silica	98.23	88.58	6.95
DBU-C12 silica	98.22	84.67	10.85
DBU-C18 silica	98.22	81.63	13.89

**Figure 4:**
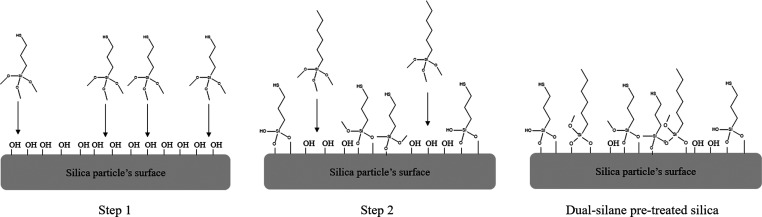
Scheme of dual-silane pretreated silica silanization process.

Scheme in Figure 4 illustrate the two-step process of dual-silane pretreated silica silanization. From the mass loss values in Table 4 we observe that most of silane is grafted during the first step. When accounting for the mass of mercaptosilane grafted on silica during the first step (10.1% mass loss), alkylsilane grafted in the second step accounts for respectively 0.7%, 2.3%, and 3.3% of the total mass loss for C3SH+C6, C3SH+C12, and C3SH+C18, respectively, which corresponds to 6.9%, 22.7%, and 32.7% of the mercaptosilane mass. Similarly, C11SH is added in the first step (15.1% of total mass) and the addition of C6 accounts for 0.6% of total mass loss and 4% of silane mass loss, which is consistent. This result comes from the fact that the mercaptosilane is added first and covers most of the surface. Alkylsilanes act as a cap and fill the remaining empty gaps of the silica surface.

**Table 4:** TGA Mass Loss Values for Dual Silane Modified Silica

sample name	residual mass at 125 °C (%)	residual mass at 600 °C (%)	mass loss (%)
HDS silica	96.9	94.6	2.3
C3SH modified silica	97.7	87.6	10.1
C3SH+C6 modified silica	98.2	87.4	10.8
C3SH+C12 modified silica	98.3	85.9	12.4
C3SH+C18 modified silica	98.3	84.9	13.4
C11SH modified silica	98.2	83.1	15.1
C11SH+C6 modified silica	98.4	82.7	15.7

**Figure 5:**
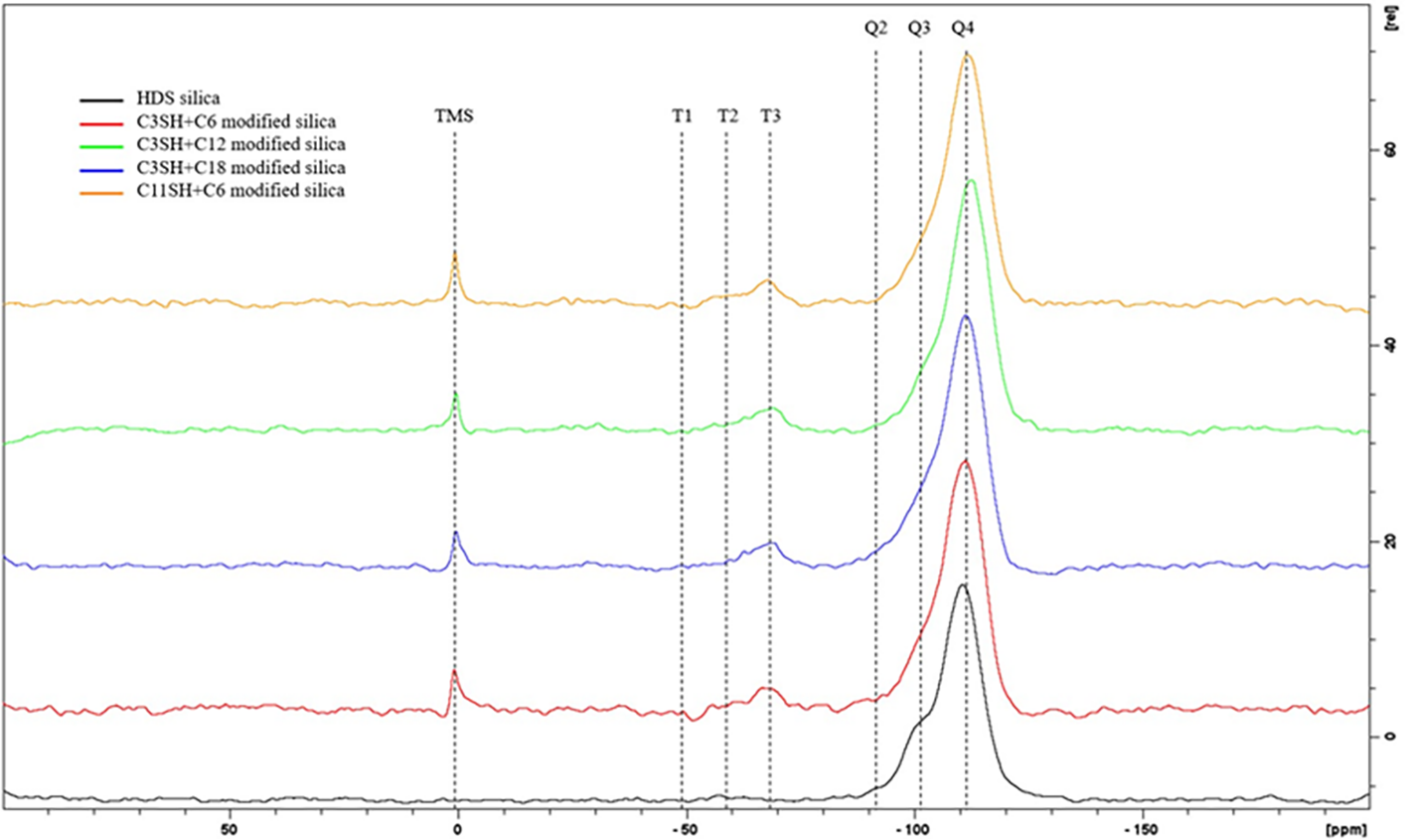
29Si ss NMR spectra of control and dual-silane premodified silica.

Figure 5 displays the results of ^29^Si ssNMR. The peak at 0 ppm corresponds to the internal standard TMS (3-(trimethylsilyl)-1-propanesulfonic acid sodium salt). Signals at −90, −100, and −110 ppm correspond to Q2, Q3, and Q4 groups. Q2 and Q3 being respectively geminal and single silanol and Q4 being the Si–O–Si bond forming the bulk of the silica. Signals at about −50, −60, and −70 ppm, respectively, correspond to T1, T2, and T3 groups. T groups are formed by the reaction of the -trimethoxysilanes on the silica surface or with another silane. This signal confirms the presence of silanes on the silica particles. It has been shown that trifunctional silanes do not react on silica surface with their three reactive groups, but with a maximum of two of them, for spatial reasons. It leads to the formation of mostly T1 and T2 groups. However, the formation of T3 groups is not impossible, and happens when silanes react together, away from the surface.^39^ We suggest that the higher content of T3 groups versus T1 and T2 results from the two-step silanization. The second silane can react with unreacted methoxy or silanol groups of first silane to yield T3 groups. The clear decrease in the Q3 peak intensity is also an evidence of the silanization reaction consuming surface hydroxyl groups. Based on the internal standard quantity and the deconvolution method, we can estimate the amount of grafted silane onto the silica. Those results are summed up in Table 5. We note a difference between samples with C3SH and the one with C11SH. With a shorter mercaptosilane as the first silane, the final silane loading is very similar. When the longer C11SH silane is used first, the final silane loading is lower. This likely comes from the higher steric hindrance displayed by the longer silane when reacting in first with the silica surface.

**Table 5:** Silane Grafting Values Based on Deconvolution of 29Si ssNMR Spectra

	HDS control	C3SH+C6	C3SH+C12	C3SH+C18	C11SH+C6
T1 (mmol)		0.00	0.00	0.00	0.00
T2 (mmol)		0.02	0.27	0.54	0.06
T3 (mmol)		0.73	0.76	0.68	0.38
total (mmol)		0.75	1.03	1.22	0.45
silane loading (mmol/g of silica)		2.48	3.32	4.05	1.49

### Dynamical Mechanical Properties

Tire tread performances, namely grip and rolling resistance, can be estimated at the material stage by testing dynamic mechanical properties of the cured rubber composite. The measurement of loss tangent tan δ on a range of temperature allows characterization of the behavior of the material.
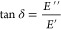
1

**Figure 6:**
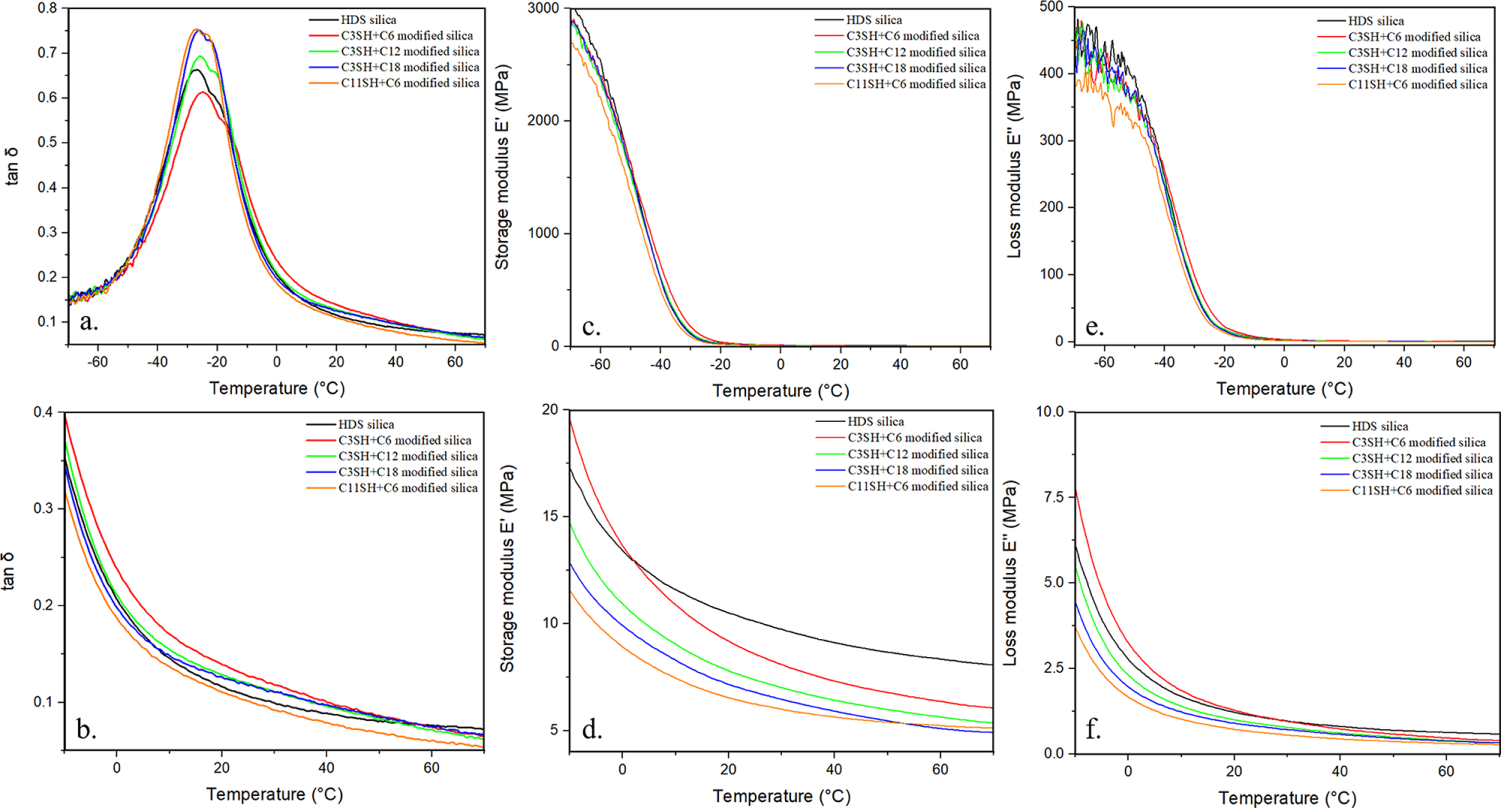
Tan (δ) (a,b), storage modulus *E*′ (c,d), and loss modulus *E*′′ (e,f) vs temperature, for various dual-silane modified silica rubber composites.

As the ratio of the loss modulus (*E*′′) and storage modulus (*E*′) in eq 1, the tan δ describes the capability of the material to dissipate energy in the form of heat through vibrational dampening. The time–temperature superposition principle allows for temperature ranges to be transposed to frequency ranges.^40^ Certain mechanical frequency ranges can be associated with mechanical solicitation of the tire tread. Therefore, tan δ at 0 °C is a good indicator for grip performances and tan δ at 60 °C a good indicator for the rolling resistance of a tire. Good grip properties are equivalent to high tan δ at 0 °C and good rolling resistance properties are equivalent to low tan δ at 60 °C.^41,42^ Ideally, one wants to maximize tan δ at 0 °C and minimize tan δ at 60 °C. The storage modulus *E*′ and tan δ of the different composite materials are represented in Figure 6, and numerical values are gathered in Table S1 of Supporting Information.

The control sample is a standard compound optimized for an 80 phr silica load and in situ silanization. Compounds with dual-silane premodified silica do not exhibit the same behavior as the control compound. Storage modulus *E*′ and tan δ decrease faster in dual-silane pretreated silica than in the control, allowing altogether for relatively higher tan δ at low temperature and low tan δ at high temperature. Samples C3SH+C6 and C3SH+C12 show improved performances for both indicators. The other two samples having slightly lower tan δ at 60 °C than the control, C11SH+C6 having a much lower tan δ than any sample. Increasing silane chain length clearly causes a decrease in storage modulus and tan δ at low temperature. The part of the curves in the −40 to −10 °C range is representative of the phase transition that the material withstands with temperature changes. Usually attributed to the glass transition temperature in polymers and rubbers, it can also display the effect of fillers.^43^ Indeed, filler particles change the polymer chain dynamic when incorporated in polymers.^27^ In our case, two overlapping peaks can be observed for all samples. The peak further on the right shifts depending on the sample. This shifting trend follows the decrease in storage modulus for all samples. It can be attributed to the increasing content of polymer chains for which the dynamic is modified by the dual-silanization. The increase of the alkyl chain length, both for the mercaptosilane and the alkylsilane, seems to lead to a decrease of the fraction of immobilized rubber. The composite sample using C3SH+C6 modified silica shows one dominant peak at lower temperature corresponding to the *T*_g_, and a second weaker relaxation process, occurring at higher temperature, probably correlated to rubber immobilized by the fillers. In view of the better dispersibility of the modified silica, the immobilization may be due to covalent linking with the SH, polymer absorbed to unshielded silica surface or perhaps to rubber which got occluded inside smaller aggregates. These two peaks seem to be separated in temperature indicating two distinct thermal transitions. The increase of the alkyl chain length for C3SH+C12 modified silica leads to an increase of the intensity of both peaks as well as to an enhanced peak-overlap of the two transitions. This may be explained by an increased amount of polymer chains involved in the relaxation processes and therefore a lower constraint exerted by the fillers on the polymer dynamics. Samples C3SH+C18 modified silica and C11SH+C6 modified silica, characterized by the longest alkyl portion in the silica-rubber coupling, exhibit very similar curves. In both cases, a sharp peak in the glass transition region is reported. In addition, an overlap of the two peaks is observed, suggesting that the dynamics of the polymer at the rubber–filler interphase and in proximity of the glass transition are very similar. Curing the green rubber compounds under a moving dye rheometer showed that the vulcanization behavior of dual-silane pretreated silica composites is completely different from that of the reference sample. These results can be seen in Figure 8.

**Figure 7:**
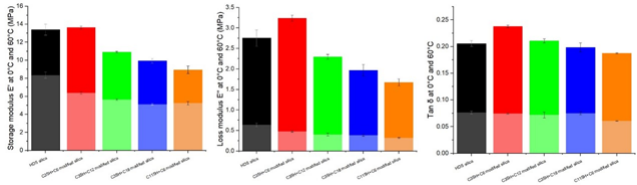
Tan δ and storage and loss modulus at 0 °C (dark colors) and at 60 °C (light colors).

**Figure 8:**
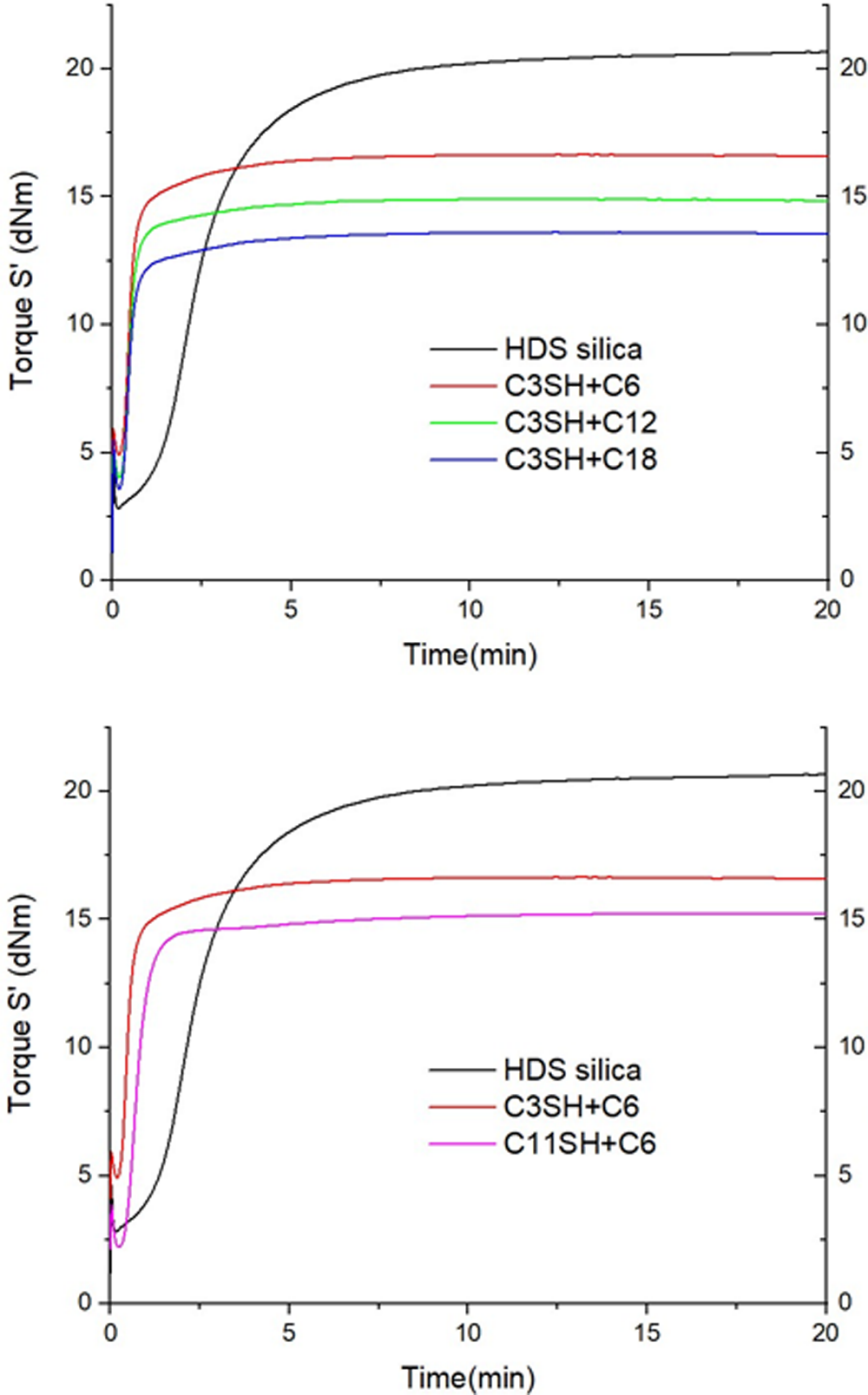
Cure behavior of green composites material measured by MDR.

Vulcanization conditions are optimized for the reference compound and have been chosen to be the same for all compounds. For dual-silane pretreated silica, vulcanization conditions are not optimal, as the scorch times are shorter, and the cure faster. No reversion is seen in any composition. The final torques are lower for all samples, suggesting that the vulcanization process may be affected, and the final cross-link density of the rubbers is lower. We suggest that the silica pretreatment and the alkylsilanes may affect the final cross-link density, leading to a lower final torque. The effect is clear for the variation in alkylsilane, where the longer is the silane, the lower is the final torque. For the longer C18 silane, the effect may be so important that it shields completely the mercaptosilane, explaining the very low storage modulus and final torque. As for the mercaptosilane variation, the initial torque difference prior to the curing of the composite is conserved and can be seen in the final torque attained in the end of the vulcanization. Both mercaptosilanes display a terminal thiol and thus show the same reactivity despite having different chain length. The sample C11SH+C6 display about the same mechanical properties as the sample C3SH+C12, where silanes are of similar length. These observations allow us to suggest that the chain length of any silane is more important than the relative size of the coupling and dispersive silanes. The initial slopes of the curves are characteristic of the cure speed. Compounds with dual-silane pretreated silica display a much faster cure behavior than the control sample. This fast cure behavior is known for premodified silica particles. Indeed, in standard tire rubber compounds, it is also intended that the silane should screen the silica particles from the polar organic molecule of curing package, such as diphenyl guanidine, in order to prevent these molecules from being excessively adsorbed on the particles and therefore not available for the vulcanization reaction. In our case because the silica is highly silanized, DPG and other vulcanizing elements are highly available in the rubber matrix and are very likely to be responsible of this fast cure.^16^

**Figure 9:**
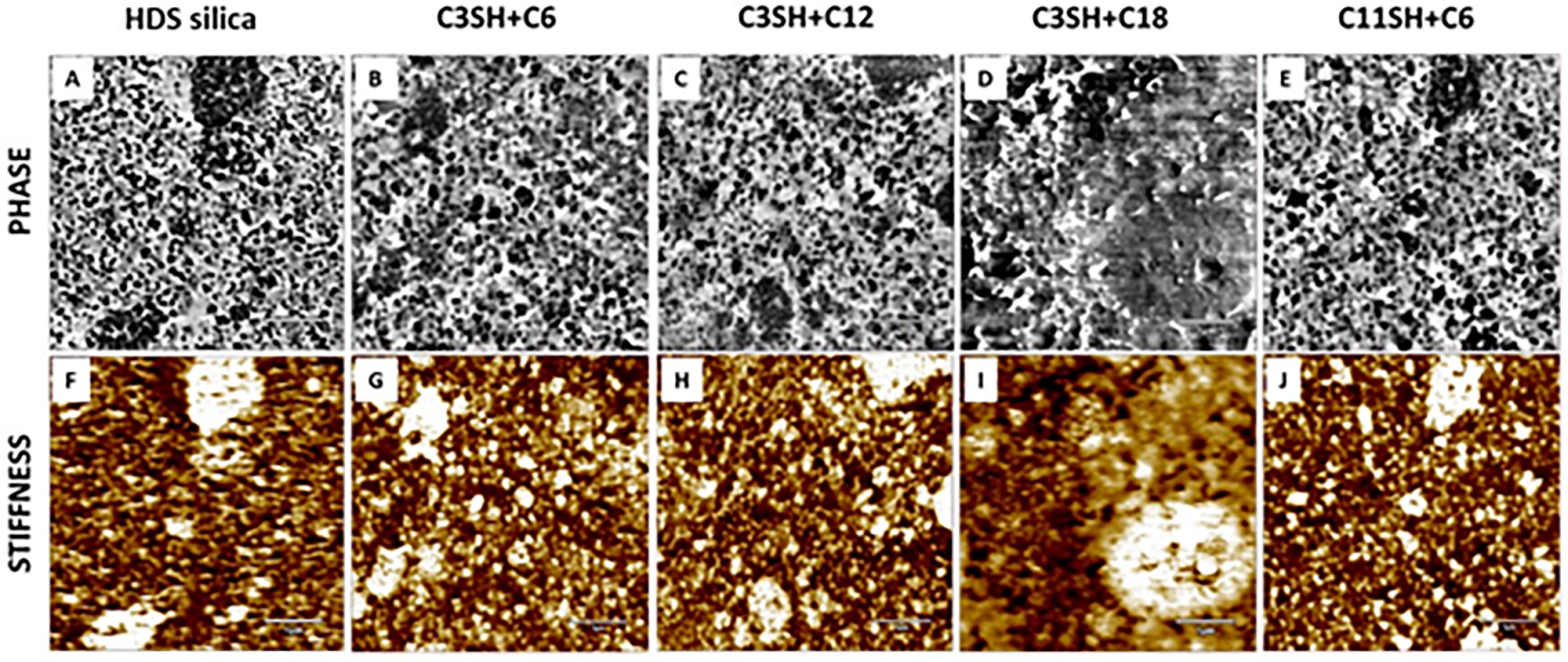
AFM phase and stiffness contrast images in areas of 5 × 5 μm^2^ of HDS silica (a,f), C3SH+C6 (b,g), C3SH+C12 (c,h), C3SH+C18 (d,i), and C11SH+C6 (e,j) composite materials.

AFM images were recorded in order to investigate the effects of the silane couplings on the materials phase and the local stiffness of the composites. This technique has the advantage of displaying a very good contrast and lateral resolution, in addition of providing complementary stiffness information. Figure 8 shows a representative AFM phase and stiffness contrast images in areas of 5 × 5 μm^2^ of the different composite materials. In the phase images, silica particles appear as dark round shape features with about 60 nm in diameter, dispersed in the rubber matrix (lighter contrast). The complementary stiffness contrast images taken simultaneously highlight the higher mechanical properties of the particles (light yellow) compared to the softer rubber matrix, confirming the attribution. Agglomerates of particles in the composites are expected, due to the high filler content in the compound, and can be seen in both phase and stiffness images, attaining even several micrometers in length (shown in the Supporting Information). The sample using C3SH+C18 silane chemistry shows large agglomerates and does not feature a similar particle distribution as the others, see Figure 9d,i.

### Atomic Force Microscopy

**Figure 10:**
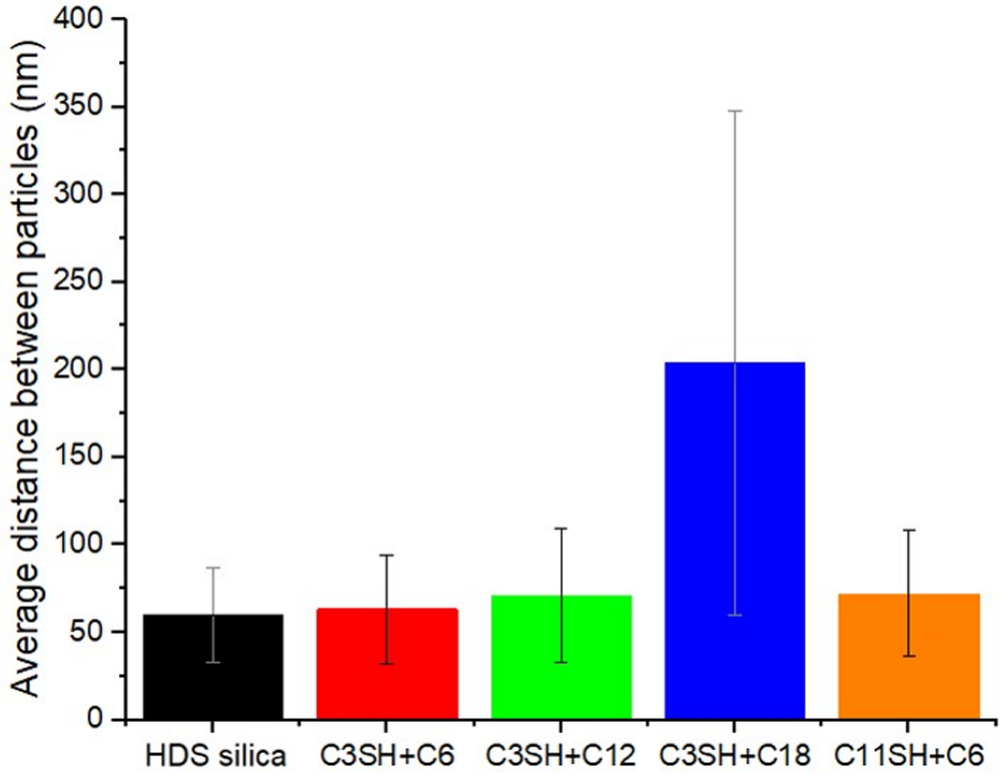
Average distance between particles (based on 10 × 10 μm images).

The average distance between particles was measured using images of similar areas in each sample for more reliable results. Focused attention should be given in zones where the presence of agglomerates is less pronounced, but since the distribution in sample C3SH+C18 was very different from the others, no reliable measurements were obtained in smaller areas. Results are shown in Table 6 and Figure 10. No significant interparticle distance difference can be observed between samples, except for C3SH+C18, for which big agglomerates form. The final mechanical properties of the material are defined by complex relations between morphology, particles sizes and distribution, but also vulcanization kinetics and interfacial interactions between components. Therefore, the dispersion of the filler alone, cannot explain the dynamical properties observed, but the higher agglomeration of the particles in sample C3SH+C18 certainly hampers the final mechanical properties of the compound.

**Table 6:** Average Distance between Particles (nm)

	average distance between particles (nm)		
sample/area	10 × 10 μm^2^	5 × 5 μm^2^	2 × 2 μm^2^
HDS control	60 ± 27	43 ± 24	28 ± 19
C3SH+C6	63 ± 31	49 ± 28	40 ± 26
C3SH+C12	71 ± 38	48 ± 27	35 ± 23
C3SH+C18	204 ± 144	120 ± 86	
C11SH+C6	72 ± 36	46 ± 26	45 ± 31

**Figure 11:**
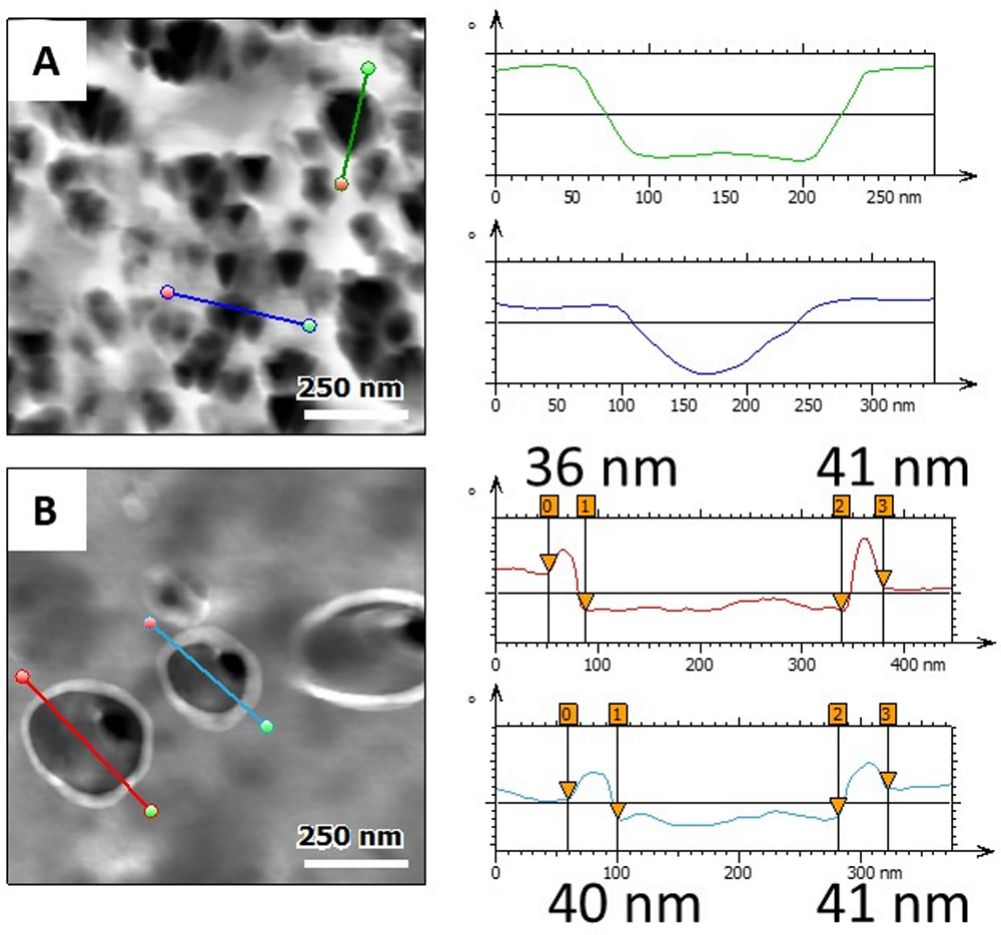
AFM phase images of areas of 1 × 1 μm^2^ and sections over silica particles of samples (A) HDS silica and (B) C3SH+C18.

In a closer look of the rubber–particle interface, we observe for the sample C3SH+C18 a 40 nm thick-layer around some nonaggregated particles, composed most likely of a mix of the silane and processing oil around the fillers. A comparison with the control sample is highlighted in Figure 11 where the control compound does not present such features. An explanation would come from the very long and therefore hydrophobic C18 silane, attracting enough oil to change the polymer chains dynamic at the interface between the filler and matrix. This effect was only observed for the longest silane, thus we suggest that the interface of the filler with the matrix can be controlled by the length of the silane and plays a great role in the dispersion of the silica and in the mechanical behavior of the composite.

## Conclusion

We synthesized dual-silane pretreated silica by preloading a basic catalyst on the particles prior to a two-step silanization. These particles were incorporated in a typical tire tread blend to investigate two major properties of tires: wet grip and rolling resistance. Short coupling silane combined with short dispersive silane demonstrated a very good compromise of properties, with high tan δ at 0 °C and low tan δ at 60 °C. We observed a clear trend in which the longer is the alkylsilane, the lower is the elastic modulus. Also, for long alkylsilane, the final torque measured by MDR is very low, suggesting a low cross-link density. We proposed that alkylsilane shields its mercaptosilane companion, decreasing its reactivity and prohibiting partially the cross-linking reaction to occur, depending on its length. It was confirmed by AFM that particles have a higher affinity to themselves with increasing silane length, thus leading to their aggregation. Even with a long mercaptosilane such as C11SH, the storage modulus and final torque are very low, suggesting that the silane is not reactive enough. The C3SH+C18 sample even displays a different phase around the filler particles, possibly due to oils attracted onto the modified particles resulting from the long silane grafted to them. This new interface is believed to deeply modify the interaction between the filler and the matrix and thus greatly impact the final composite mechanical properties. Overall, dual-silane pretreated silica have a different behavior compared to the control filler: higher tan δ at low temperature and lower tan δ at higher temperature are achieved for the shorter alkylsilane C6 and C12 when paired with C3SH. Pretreatment of the silica before incorporation in rubber allows for more control over silane grafting on the particles, as well as avoiding the production of subsequent alcohols resulting from the silanization during rubber mixing. The use of two silanes shows new behavior of the nanocomposites as well as specific limits: a short silanes combination brings better mechanical properties, namely higher tan δ at 0 °C suggesting better wet grip, but longer alkylsilanes, especially C18, fail to ensure reinforcement and lead to lower performance with regards to tire-tread properties requirements. The longer is the alkyl chain, the more intersilane interactions via weak van der Waals forces are enhanced, creating a packed layer of alkyl chains around the silica particles and resulting in poor interaction with the polymer matrix. Additionally, long alkyl chains diminish the accessibility of thiol grafted onto silica for vulcanization and cross-linking, leading to even less interaction between silica and the matrix and resulting in insufficient reinforcement. As tan δ alone cannot describe the entire behavior of the rubber of a tire, further studies should focus on testing such dual-silane pretreated silicas on a large scale volume to test the subsequent ultimate properties of a real tire.

## Supporting Information Available

Additional silica SEM images, silica infrared spectrum, DMA data table, and AFM micrograph (PDF)

## Abbreviations

MDR: moving dye rheometer
TESPT: bis-triethoxypropylsilyltretrasulfide
HDS: high dispersibility silica
C3SH: 3-mercaptopropyltrimethoxysilane
C11SH: 11-mercaptoundecyltrimethoysilane
C6: hexyltrimethoxysilane
C12: dodecyltrimethoxysilane
C18: octadecyltrimethoxysilane
THF: tetrahydrofuran
DBU: 1,5-diazabicyclo[5.4.0]undec-5-ene
SBR: styrene–butadiene rubber
BR: butadiene rubber
TDAE oil: treated distillate aromatic extracted oil
6-PPD: *N*-(1,3-dimethylbutyl)-*N*′-phenyl-p-phenylenediamine
MBT: 2-mercaptobenzothaizole
DPG: diphenylguanidine
CBS: *N*-cyclohexyl-2-benzothiazolesulfenamide
TGA: thermogravimetric analysis
^29^Si MAS ssNMR: silicon 29 magic angle spinning solid state nuclear magnetic resonance spectrometry
DMA: dynamic mechanical analysis
AFM: atomic force microscopy
TMS: 3-(Trimethylsilyl)-1-propanesulfonic acid sodium salt
