# Supplementary material for: Dual-Silane Premodified Silica Nanoparticles—Synthesis and Interplay between Chemical, Mechanical, and Curing Properties of Silica–Rubber Nanocomposites: Application to Tire Tread Compounds
Source: ACS Omega. 2022 May 18;7(21):17692–702. doi: 10.1021/acsomega.2c00665 (PMC9161251; doi:10.1021/acsomega.2c00665)
Supplement: Supplementary file 1 — ao2c00665_si_001.pdf [file ao2c00665_si_001.pdf]

Dual-silane pre-modified silica nanoparticles – Synthesis and interplay between chemistry, mechanical and curing properties of silica-rubber nanocomposites: Application to tire tread compounds

*Enzo Moretto[1], João P. C. Fernandes[1], Mariapaola Staropoli[1], Vincent Rogé[1], Pascal Steiner[2], Benoît Duez[2], Damien Lenoble[1], Jean-Sébastien Thomann[1]**

[1] Luxembourg Institute of Science and Technology, MRT department; 41 rue du Brill, L-4422 Belvaux, Luxembourg; jean-sebastien.thomann@list.lu

[2] Goodyear S.A, Avenue Gordon Smith, L-7750 Colmar-Berg, Luxembourg; benoit_duez@goodyear.com

Correspondence: jean-sebastien.thomann@list.lu

KEYWORDS: Silica, silane, silanization, filler interphase characterization, nanocomposite, tire tread.

Supporting Information

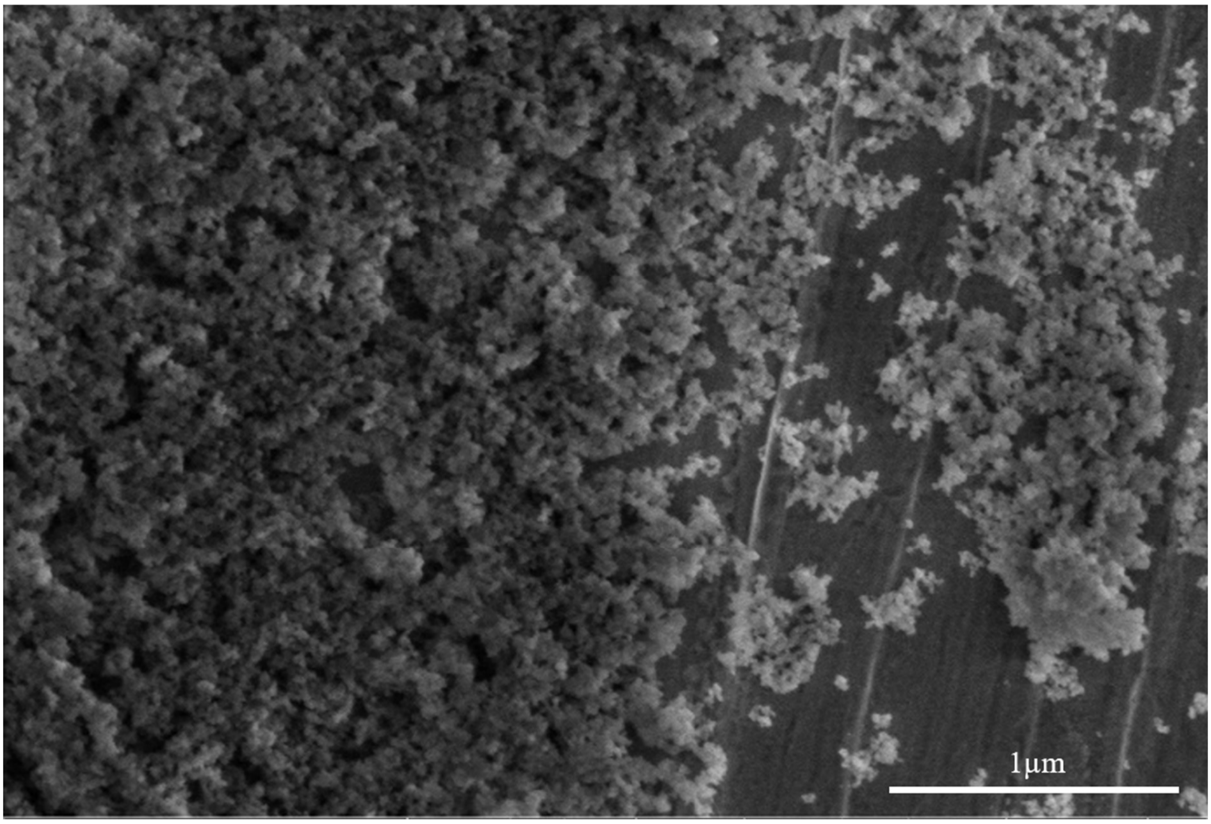

Figure S 1 - HDS precipitated silica under scanning electron microscope

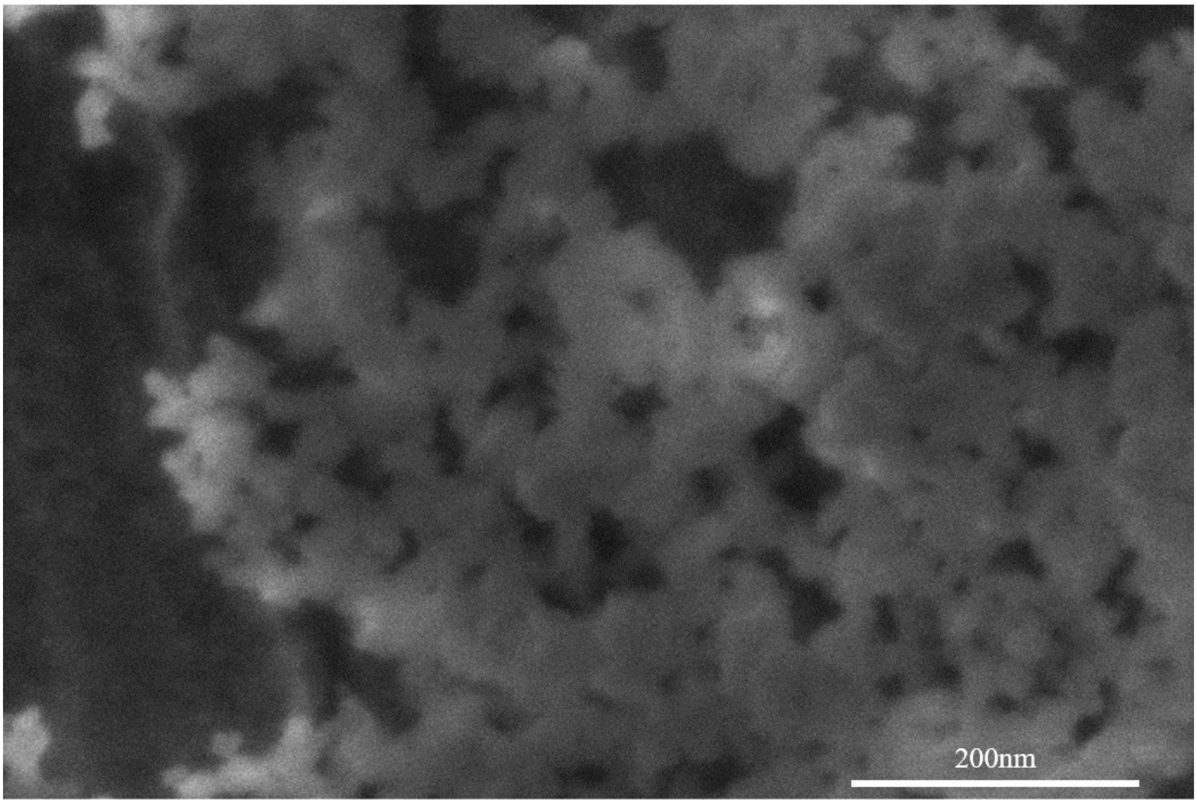

Figure S 2 - HDS precipitated silica under scanning electron microscope

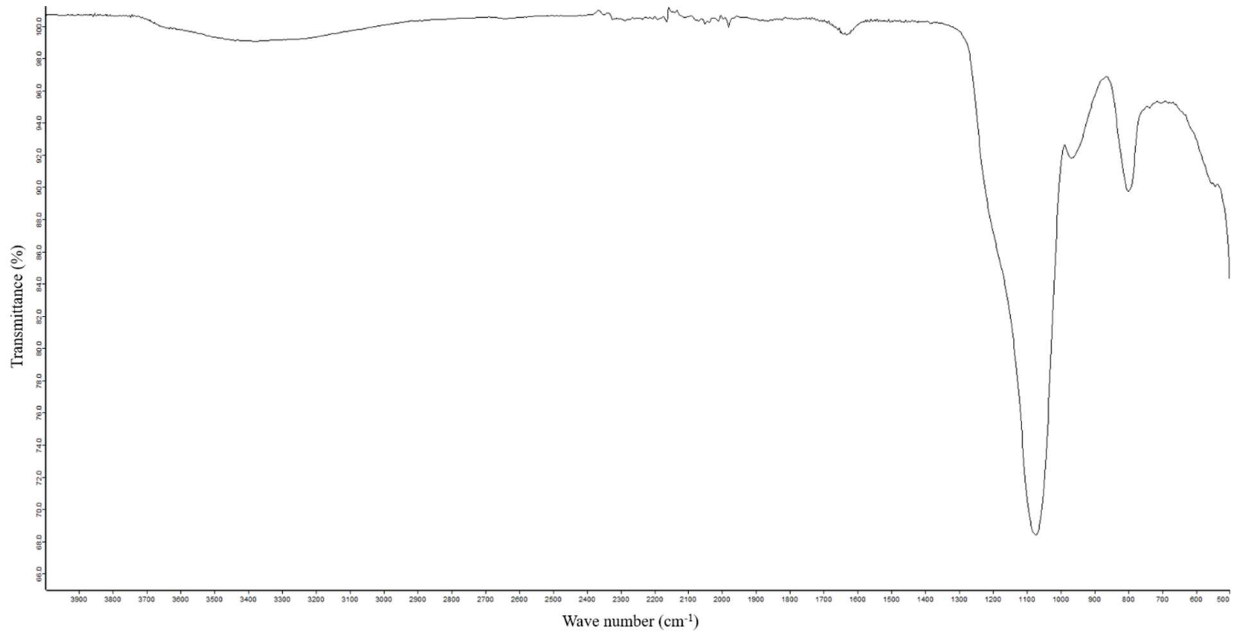

Figure S 3 - ATR infrared spectrum of HDS precipitated silica

Si-OH adsorption band : 3000-3800cm^{-1}

Si-O-Si adsorption band : 470, 800 and 1070cm^{-1}

Table S 1 - Storage modulus E', loss modulus E'' and tan(δ) of dual-silane silica rubber composite

		Storage modulus E’		Loss modulus E’’		Tan δ	
		at 0°C (MPa)	at 60°C (MPa)	at 0°C (MPa)	at 60°C (MPa)	at 0°C	at 60°C
HDS control		13.39±0.60	8.35±0.34	2.75±0.19	0.63±0.04	0.205±0.005	0.076±0.002
C3SH	C6	13.63±0.16	6.37±0.12	3.23±0.07	0.47±0.01	0.237±0.002	0.074±0.001
C3SH	C12	10.90±0.075	5.63±0.059	2.29±0.05	0.40±0.03	0.210±0.003	0.070±0.005
C3SH	C18	9.963±0.27	5.10±0.091	1.96±0.13	0.38±0.01	0.198±0.008	0.074±0.002
C11SH	C6	8.933±0.41	5.242±019	1.67±0.08	0.31±0.01	0.187±0.001	0.060±0.003

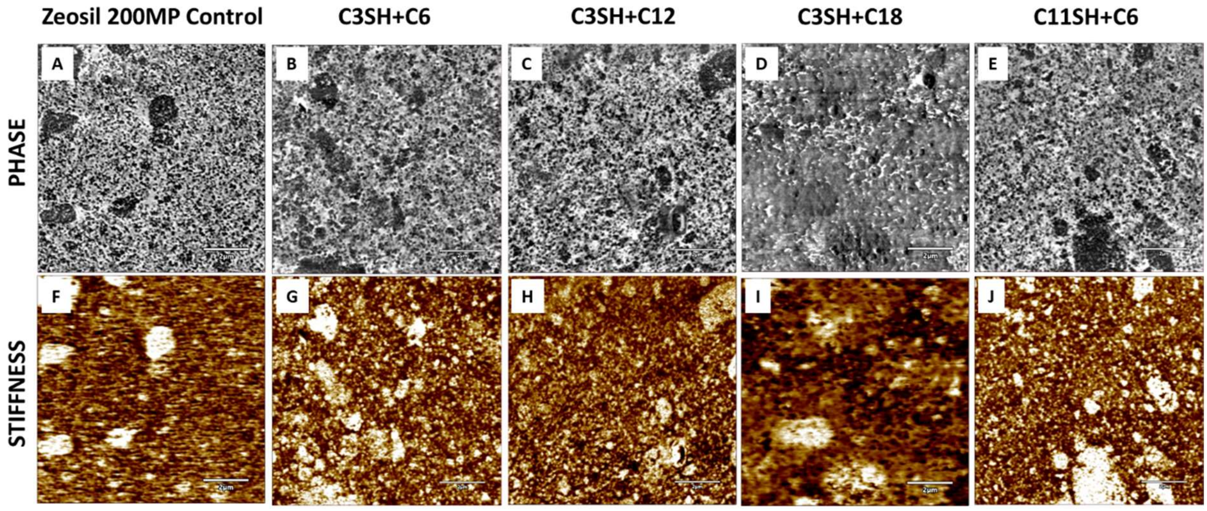

Figure S 4 - AFM phase and stiffness contrast images in areas of 10x10 μm² of the different composite materials.

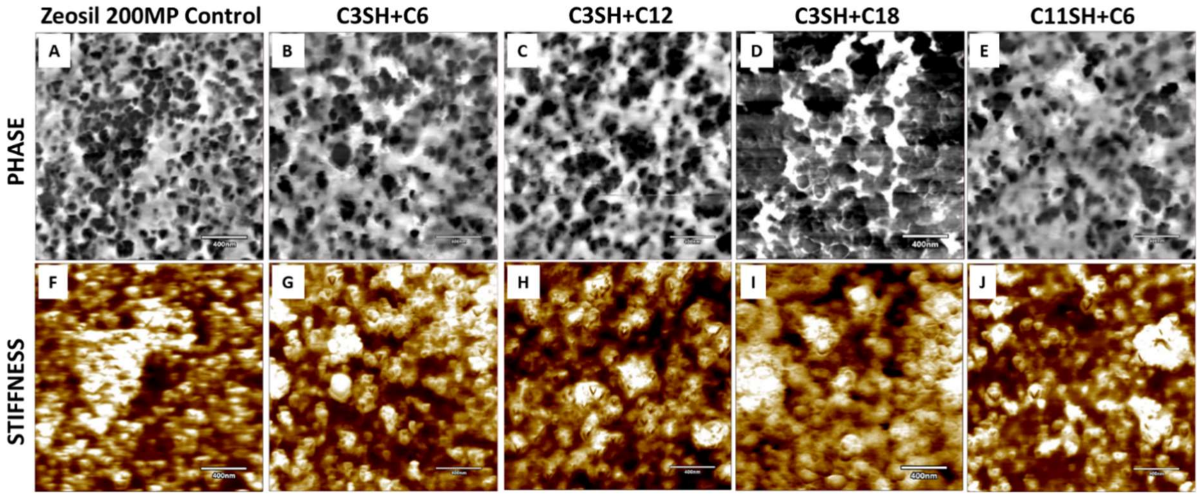

Figure S 5 - AFM phase and stiffness contrast images in areas of 2x2 μm² of the different composite materials
